# Urinary Volatilomic Signatures for Non-Invasive Detection of Lung Cancer: A HS-SPME/GC-MS Proof-of-Concept Study

**DOI:** 10.3390/ijms27020982

**Published:** 2026-01-19

**Authors:** Patrícia Sousa, Pedro H. Berenguer, Catarina Luís, José S. Câmara, Rosa Perestrelo

**Affiliations:** 1 CQM—Centro de Química da Madeira, Universidade da Madeira, Campus da Penteada, 9020-105 Funchal, Portugal; patricia.sousa@staff.uma.pt (P.S.); cgsluis@staff.uma.pt (C.L.); jsc@staff.uma.pt (J.S.C.); 2 Centro de Investigação Dra. Maria Isabel Mendonça, Hospital Dr. Nélio Mendonça, SESARAM, EPERAM, Avenida Luís de Camões, n^o^57, 9004-514 Funchal, Portugal; 3 Faculdade de Ciências da Vida, Universidade da Madeira, Campus da Penteada, 9020-105 Funchal, Portugal; 4 Departamento de Química, Faculdade de Ciências Exatas e Engenharia, Universidade da Madeira, Campus da Penteada, 9020-105 Funchal, Portugal

**Keywords:** lung cancer, urine, volatilomic signature, multivariate analysis, biomarkers

## Abstract

Lung cancer (LC) remains the leading cause of cancer-related death worldwide, largely due to late-stage diagnosis and the limited performance of current screening strategies. In this preliminary study, headspace solid-phase microextraction coupled with gas chromatography–mass spectrometry (HS-SPME/GC-MS) was used to comprehensively characterize the urinary volatilome of LC patients and healthy controls (HCs), with the dual aim of defining an LC-associated volatilomic signature and identifying volatile organic metabolites (VOMs) with discriminatory potential. A total of 56 VOMs spanning multiple chemical classes were identified, revealing a distinct metabolic footprint between groups. LC patients exhibited markedly increased levels of terpenoids and aldehydes, consistent with heightened oxidative stress, including lipid peroxidation, and perturbed metabolic pathways, whereas HCs showed a predominance of sulphur-containing compounds and volatile phenols, likely reflecting active sulphur amino acid metabolism and/or microbial-derived processes. Multivariate modelling using partial least squares-discriminant analysis (PLS-DA, R^2^ = 0.961; Q^2^ = 0.941; *p* < 0.001), supported by hierarchical clustering, demonstrated robust and clearly separated group stratification. Among the detected VOMs, octanal, dehydro-p-cymene, 2,6-dimethyl-7-octen-2-ol and 3,7-dimethyl-3-octanol displayed the highest discriminative power, emerging as promising candidate urinary biomarkers of LC. These findings provide proof-of-concept that HS-SPME/GC-MS-based urinary volatilomic profiling can capture disease-specific molecular signatures and may serve as a non-invasive approach to support the early detection of LC, warranting validation in independent cohorts and integration within future multi-omics diagnostic frameworks.

## 1. Introduction

Cancer is the second leading cause of death worldwide, next to cardiovascular diseases, with an estimated 13.1 million deaths in 2030, as estimated by the World Health Organization (WHO) [1]. Lung cancer (LC) is the second most common neoplasms and the leading cause of mortality worldwide, representing 18.7% of all cancer deaths [2,3]. The highest incidence rates worldwide are observed in Micronesia/Polynesia, Eastern and Southern Europe, Eastern Asia, and rates are usually lower for Africa, except North and South Africa [4].

In Portugal, LC is among the most common and lethal malignancies, with an estimated incidence and mortality rate of 18.5/100,000 [2,3]. Prostate, colorectal and LC are the most frequent cancers in men, and breast, colorectal and lung in women. While there has been a general decline in cancer mortality, deaths by LC in Portuguese females increased by about 21% in the last decade, reflecting shifts in behavioural risk factors [3]. These data underscore the need of implementing an effective screening strategy among risk groups, stronger tobacco control policies, and improved equitable access to cancer care for the Portuguese population [5].

The late onset of symptoms and the fact that over 75% of LCs are confirmed at advanced stages make early detection the main issue [4]. Advances in immunotherapies and targeted therapies have improved the prognosis for certain patient subgroups, but early detection is believed to be more effective in reducing mortality. Physical examination, imaging (e.g., chest radiographs, computed tomography and magnetic resonance imaging), biopsy for histopathological assessment and molecular profiling, including identification of specific genetic mutations or biomarkers, cancer subtype identification, non-small cell LC vs. small cell LC, and staging collectively guide treatment selection [6,7]. Therapeutics are determined by the cancer subtype, stage, patient comorbidities, and include surgery, radiotherapy, immunotherapy, targeted therapy and chemotherapy. Treatment success lies in early diagnosis, which is often not achievable as clinical symptoms may be absent or subtle in the initial stages of disease.

In recent years, cancer control efforts have shown limited effectiveness, imposing tremendous pressure on patients, society and healthcare systems. In response, OMICS science, which encompasses the study of biological molecules at the genomic, transcriptomic, proteomic, lipidomic and metabolomic levels, offers significant contributions to the understanding of tumour behaviour. In this field, the metabolic characterisation of human biofluids [8], including plasma [9], serum [10], sweat [11], saliva [12] and urine [13], has become increasingly important, particularly in LC, since intrinsic differences in the metabolism of cancer cells compared to normal cells give rise to metabolomic signatures that are useful for distinguishing cancer patients from healthy controls (HCs), thus allowing the identification of highly sensitive and specific biomarkers. In this way, several research groups have demonstrated the effectiveness of such approaches through the analysis of volatile organic metabolites (VOMs) that signal the metabolic process of cancer cells [14,15,16,17]. Cancer development is associated with cellular oxidative stress, which increases the expression of cytochrome P-450 oxidase enzymes and ultimately affects the levels of VOMs in biological matrices. These VOMs, generated by lipid peroxidation and further metabolised by the cytochrome P-450 enzyme, are readily distinguishable and provide a unique “metabolic fingerprint” [15]. VOMs are promising biomarkers for the early detection of LC, as they directly reflect the metabolic activity of cancer cells. To establish the volatilomic signature and, consequently, identify possible volatile biomarkers of LC in biofluids, various analytical approaches have been proposed, mainly using the gold standard of solid-phase microextraction combined with gas chromatography-mass spectrometry (SPME/GC-MS). In this context, urine has already been highlighted as one of the most promising matrices for diagnosis [18], since most VOMs are present in this biofluid and contain crucial information about the clinical condition of patients.

However, despite being non-invasive and cost-effective, the identification of VOMs in urine faces challenges such as a lack of standardisation in studies, complexity of data analysis, and confounding by variables associated with genetics and lifestyle. Despite these problems, the identification of individual VOMs offers a potentially beneficial means of early and accurate diagnosis of diseases such as LC. This potential has indeed been supported by recent studies carried out by Rubio-Sánchez et al. [16] and Taunk et al. [14], both of which reported promising results in the use of urinary VOMs as potential LC biomarkers through SPME/GC-MS analysis. The results highlight the critical necessity for continued research and the establishment of standardized diagnostic frameworks to facilitate LC early detection. Further potential of VOMs has been shown in the diagnosis of many other cancers, including breast, colorectal, bladder and oesophageal, proving its cross-sectional nature and robustness [19]. In bladder cancer, urinary VOMs have demonstrated strong diagnostic potential. Gas chromatography—ion mobility spectrometry (GC-IMS)-based models achieved accuracies exceeding 90% [17], and HS-SPME/GC-MS approaches identified robust metabolic signatures capable of discriminating patients from HCs with high diagnostic accuracy (area under the curve (AUC) > 0.80) [20,21]. Similar observations were made in colorectal cancer, where VOMs like *p*-cresol and 3(4H)-dibenzofuranone were detected in faeces as biomarkers distinguishing adenoma carriers from HCs, further reinforcing the promise of this route in the early detection of premalignant lesions [22]. Similar evidence was found in oesophageal cancer, where urinary volatilomics analysis on 162 subjects, using GC-IMS combined with machine learning, showed eight VOMs with excellent discrimination abilities (AUC = 0.874). These include 2,3-butanediol, 2-acetylfuran, dimethyl trisulfide, methyl 2-methylbutanoate, methyl decanoate, (*E*)-ethyl 2-hexenoate, 2-isopropyl-3-methoxypyrazine, and cyclohexanone D. Their levels were significantly altered and might serve as potential biomarkers [23]. Likewise, the HS-SPME/GC-MS analysis of tumour and normal gastric tissues [23] demonstrated a consistent alteration in the levels of pyridine, hexanal, and nonanal in this study. These changes indicate that such metabolites can be further explored as potential molecular targets in the development of early detection breath- or urine-based tests for disease.

In this context, the present study aims to characterise, for the first time in detail, the urinary volatilomic signature associated with LC using a standardised HS-SPME/GC-MS workflow, and to identify VOMs capable of discriminating patients from HCs. While most volatilomic investigations in LC have focused on breath, plasma or serum, urine remains comparatively underexplored despite its clinical advantages as a non-invasively obtained, metabolite-rich biofluid. By profiling a broad spectrum of urinary VOMs and applying robust multivariate statistical modelling, this study seeks not only to expand current knowledge on LC-related metabolic alterations but also to contribute novel candidate biomarkers and methodological insights that address key gaps in the existing volatilomics literature.

## 2. Results and Discussion

### 2.1. Volatilomic Signature

**Figure 1:**
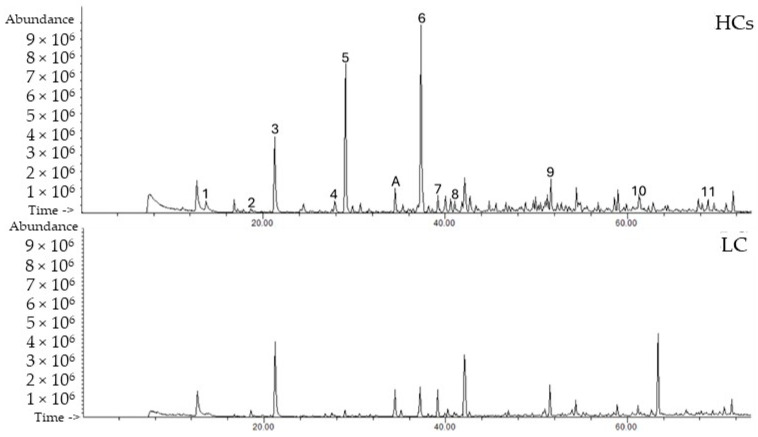
Representative chromatograms of the LC patients and HCs using HS-SPME/GC-MS. Peak number identification: (1) 2,5-dimethylfuran; (2) dimethyl disulfide; (3) 4-heptanone; (4) δ-terpinene; (5) p-cymene; (6) dehydro-p-cymene; (7) 2-ethyl-1-hexanol; (8) theaspirane; (9) dehydro-ar-ionene; (10) 3,4-dehydro-β-ionone; (11) cadalene. (A) 3-octanol, IS.

VOMs have been described as a promising class of biomarkers for specific diseases through the definition of volatilomic signatures. These sets of VOMs have the potential to be used in early detection, as diagnostic tools, and to monitor disease progression and therapy efficacy [14,16]. Following HS-SPME/GC-MS analysis of urine samples from the 45 recruited participants, distinct chromatographic profiles were observed for LC patients and HCs (Figure 1, Table 1). At the time of urine collection, the majority of lung cancer (LC) patients were treatment-naïve. A small subset had received anticancer therapy prior to sampling: three patients underwent systemic pharmacological treatment (two receiving chemotherapy and one receiving combined chemotherapy and immunotherapy), one patient underwent surgical resection followed by radiotherapy, and one patient underwent surgery alone. The remaining LC patients had not received any form of anticancer treatment before urine collection. Detailed information regarding tumour stage, morphology, and treatment status for each patient is provided in Table S1.

Fifty-six VOMs were identified in the samples analysed, belonging to different chemical families, which included 16 terpenoids, eight furanic compounds, three volatile phenols, five ketones, four sulphur-containing compounds, five aldehydes, three norisoprenoids, three alcohols, and nine others (including four benzene derivatives, two polycyclic aromatic hydrocarbons, one lactone, and one ester), as shown in Table 1.

Each group of samples shows differences in the relative areas for the different chemical families (Table 1). Urinary excretion is one of the ways in which the body detoxifies VOMs, and thus, organic acids are eliminated from the body. The human urinary profile changes over time due to various factors such as bacterial activity, metabolism, pH variations or degradation of urinary constituents, and is influenced by various external factors such as health status, dietary habits, physical stress and the environment, which together with endogenous compounds contribute to the volatilomic biosignature of individuals. Although these factors play a role, human metabolism is highly complex, and the onset of cancer complicates the task of understanding all the metabolic processes that may contribute to the change or persistence of specific VOMs [14]. Therefore, it is extremely important to establish a relationship between the VOMs found in samples and their potential endogenous origin. To date, the source of most VOMs has yet to be defined [24]. In many cases, the same metabolite can be obtained both endogenously and exogenously, making it a very challenging process to understand its metabolic pathway [14]. Thus, the recognition of the metabolic pathways leading to the production and clearance of VOMs will provide new insights into the biochemistry of cancer [24].

**Figure 2:**
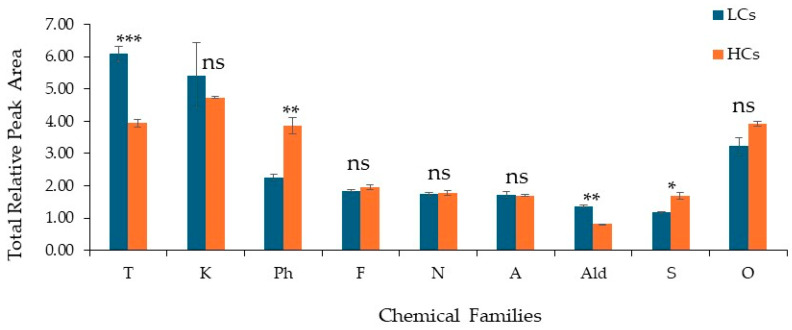
Distribution of the chemical families identified in the studied groups, LC patients (n = 21) and HCs (n = 24). Legend: A: alcohols; Ald: aldehydes; F: furanic compounds; K: ketones; N: norisoprenoids; S: sulphur-containing compounds; T: terpenes; Ph: volatile phenols; O: others. Statistical differences between the LC and HC groups were assessed using an unpaired Student’s *t*-test (Welch correction). Significance levels are indicated as * *p* < 0.05, ** *p* < 0.01 and *** *p* < 0.001; ns: not significant.

The present study analysed and compared urinary profiles of VOMs in LC patients and HCs, identifying 56 VOMs from different chemical families. Significant differences in the relative proportions of the identified chemical families were observed using the mean of total relative peak areas (RPA) as a semi-quantitative measure. Terpenoids were the most prevalent chemical family in both groups, followed by ketones, volatile compounds, furanic compounds, norisoprenoids, alcohols, aldehydes, and sulphur compounds (Figure 2). Among the analysed chemical families, significant differences between LC and HC groups were observed for sulphur compounds (S), terpenoids, aldehydes (Ald) and volatile phenols (VP) (*p* < 0.05). Specifically, terpenoids and aldehydes were present at higher relative abundances in LC samples, whereas volatile phenols and sulphur compounds showed significantly higher levels in HC samples. On the other hand, furanic compounds, ketones, alcohols, norisoprenoids and other chemical families did not present statistically significant differences between the groups (*p* > 0.05).

Pharmacological treatment may influence the urinary volatilomic signature by altering systemic metabolism, inflammatory status, and drug biotransformation pathways. In the present study, a small subset of LC patients had received systemic therapy prior to sample collection. To mitigate potential treatment-related variability, only VOMs detected in at least 85% of samples were included in the statistical analysis, prioritising robust and consistently detected VOMs. Nonetheless, the impact of anticancer therapy on urinary VOM composition cannot be fully excluded and represents a limitation of this study. Future work should address this limitation using larger, clinically stratified cohorts, with treatment-naïve and treated patients analysed separately.

In LC patients, terpenoids were the most abundant compound family, forming most of the urinary volatilomic signature, consistent with recent studies that have identified limonene, α-pinene, β-pinene and *p*-cymene as potential volatile biomarkers of LC [25,26]. The elevated levels of terpenoids in urine samples characterised as LC suggest an increase in the metabolism of fatty acids and carotenoids, resulting in the accumulation of isoprenoid intermediates. These results also align with previous findings showing considerably higher concentrations of terpene compounds in a tumour context, reflecting chronic inflammation and oxidative damage within the tumoral microenvironment [14,27].

Elevated levels of the aldehydes and ketones were also detected in LC. Hexanal, octanal and 2-heptanone were identified as indicators of lipid peroxidation and cellular membrane degradation [28,29]. In contrast, sulphur chemical families (e.g., dimethyl disulfide, methanethiol, dimethyl trisulfide) were increased in HCs, which is indicative of functional sulphur biotransformation in HCs. Furthermore, the reduced abundance in LC patients could be related to the increased metabolism of sulphur-containing amino acids (e.g., methionine, cysteine) by tumours, changes in *trans*-sulphuration pathways, and intestinal isolate or microbiota dysbiosis, as reported by Zhang et al. [30] and Porto-Figueira et al. [31].

Although furanic compounds tended to be more prevalent in the HC group, RPA revealed no statistically significant differences between LC and HC (*p* > 0.05), with 2,5-dimethylfuran, 2-methylfuran, and 2-ethyl-5-methylfuran being the predominant VOMs. These VOMs are related to lipid peroxidation and mitochondrial dysfunction. According to studies linking elevated furan levels to the oxidative metabolism of unsaturated fatty acids and to lipid peroxidation, their increase is indicative of enhanced oxidative stress and accelerated tumour metabolic activity [32,33].

Similarly, volatile phenols showed significant RPA in HCs (*p* < 0.01), which may be representative of exogenous contributions (e.g., diet, environmental exposure, smoking), as well as physiological bacterial metabolism present in HCs. There is conflicting literature that does not show that HCs have increased values of these VOMs compared to LC patients. The current results are heterogeneous and dependent on the biological matrix and type of neoplasia [19].

### 2.2. Statistical Analysis

**Figure 3:**
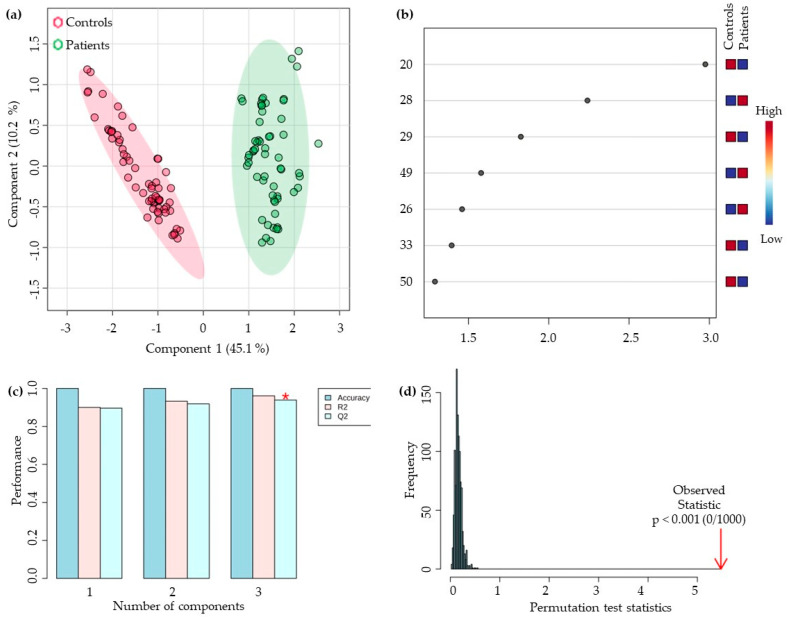
(**a**) Partial least-squares discriminant analysis (PLS-DA) score plot; (**b**) Variables of importance in projection (VIP) scores plot; (**c**) 10-fold CV performance of the PLS-DA classification using a different number of components (* means best Q^2^ value, the best classifier) and (**d**) PLS-DA model validation by permutation tests based on 1000 permutations of the VOMs obtained by GC-MS of the urine samples from the groups under study. Peak number identification: (20) octanal, (28) dehydro-p-cymene, (29) 2,6-dimethyl-7-octen-2-ol, (49) 2-methoxyphenol, (26) 3,7-dimethyl-3-octanol, (33) 2-acetylfuran, and (50) phenol.

After characterising the urinary volatilomic signatures of the LC and HC groups, the dataset was processed using statistical approaches to evaluate discriminatory patterns between groups. To ensure model robustness, only VOMs detected in at least 85% of samples (n = 45) were included in the analysis. Prior to modelling, data were normalised to reduce heteroscedasticity and improve comparability across samples, thereby removing redundant variables that did not contribute to group differentiation. Of the 56 VOMs retained, 44 were statistically significant (*p* < 0.001). PLS-DA multivariate pattern recognition, the information contained in the VOM fingerprint is used as several variables to visualize group trends and clustering patterns depending upon the separations among sample sets. The statistical model obtained separates the LC and HCs urine samples into two clusters corresponding to the tumour and non-tumour samples analysed (Figure 3a), confirming these discriminant trends with good discrimination between groups (component 1 explaining 45.1% variance and component 2 explaining 10.2%).

The VIP scores plot describes the relative contribution of the VOMs to the variance between the two groups. In addition, seven VOMs were identified as being differentially expressed with VIP higher than 1 (Figure 3b), several of which have already been reported as discriminants in different clinical conditions. Octanal, like other aldehydes, has been reported by several authors as a discriminating metabolite for LC [34]. Jia et al. [35] identified significantly higher levels of this aldehyde in the exhaled air of patients with LC compared to HCs, indicating oxidative dysfunctions associated with LC metabolism. Interestingly, the present study observed that octanal (#20) had higher mean levels in HCs than in LC, an observation that contrasts with the typical signatures described in the literature [36]. This apparent lower abundance of octanal in LC may reflect a metabolic adaptation to chronic oxidative stress, suggesting that as the tumour progresses, phenomena of consumption or accelerated catabolism of aldehydes occur [37]. It is important to note that the directionality of VOM alterations is not universal across cancer types, biological matrices, or analytical platforms. In some oncological contexts, several VOMs are decreased in patients relative to HCs [38], underscoring the metabolic complexity of tumour biology. Conversely, 2-methoxyphenol (#49) presented higher mean levels in LCs than in HCs; however, this compound is strongly influenced by exogenous factors such as diet, smoking, and environmental exposure, which may explain its elevated levels in LCs [39]. Therefore, the patterns observed here likely reflect metabolic specificities of LC in combination with environmental, behavioural, and methodological variability. These considerations highlight the need for validation in independent cohorts with stringent control of exogenous exposures and analytical standardisation. In the case of the metabolite dehydro-*p*-cymene (#28), a structural derivative of the cymene family, higher levels were observed in the LC group. This aromatic monoterpene has been repeatedly identified in volatilomic signatures associated with LC, along with *p*-cymene and *o*-cymene [40,41]. Although there are still a few studies confirming its biomarker potential, Al-Kateb et al. [42] reported dehydro-*p*-cymene among VOMs of diagnostic interest, observing higher levels in LC patients and a possible association with environmental pollutants. Thus, the observed increase is biologically plausible, reflecting the role of monoterpenes in tumour metabolism and the oxidative dysregulation typical of the disease [27]. In turn, 2,6-dimethyl-7-octen-2-ol and 3,7-dimethyl-3-octanol were observed as discriminating biomarkers in urinary and exhaled air volatilomic patterns in prostate and lung cancer, with higher levels in patient groups [26].

To confirm the robustness and validity of the statistical model obtained, a random permutation test with 1000 permutations (Figure 3c) and 10-fold CV cross-validation (Figure 3d) were performed with the PLS-DA model. The model showed excellent fit (R^2^ = 0.961) and high predictive power (Q^2^ = 0.941) in the three significant components, showing that the model is not overfitted and has good predictive power to distinguish between the studied groups (R^2^ − Q^2^ = 0.02). The permutation test confirmed the statistical significance of the discrimination between classes (*p* < 0.001), demonstrating that the model was not overfit. Thus, the PLS-DA model reveals high robustness and strong predictive power, allowing statistically significant distinction between the groups studied and supporting its reliability for further biological interpretation.

**Figure 4:**
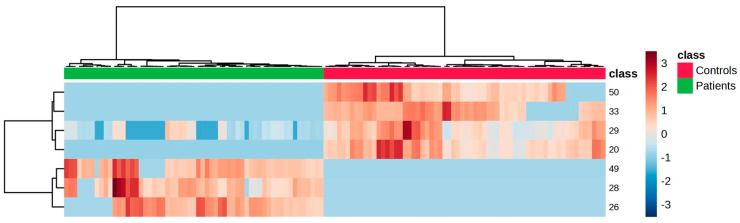
Clustering results shown as a heatmap illustrate the relative peak area of the urinary volatile organic metabolites identified in each sample. Columns correspond to the LC (green) and HC (red) sample groups, whereas rows correspond to the most relevant VOMs detected. The colour of the cells corresponds to the normalised peak areas of the VOMs (minimum −3, dark blue; maximum +3, dark red). Peak number identification: (20) octanal, (28) dehydro-p-cymene, (29) 2,6-dimethyl-7-octen-2-ol, (49) 2-methoxyphenol, (26) 3,7-dimethyl-3-octanol, (33) 2-acetylfuran, and (50) phenol.

Finally, hierarchical analysis was performed based on the VOMs selected in the PLS-DA model, using the Ward linkage method and Euclidean distance as a similarity metric. The heatmap created using Pearson’s correlation for the VOMs with VIP scores higher than 1.0 is shown in Figure 4. It is possible to observe a clear separation between the two groups, reflected by the formation of two large, distinct clusters. The samples from the HCs predominantly show negative values (blue shades), while the LCs show higher values (red shades), indicating systematic differences regarding the relative expression of the selected variables. This pattern reinforces the statistical model’s discriminative ability and highlights potential biomarkers between groups. It should be noted, however, that this is only a preliminary study involving a limited number of samples. Larger studies with higher sample sizes and independent patient cohorts are needed to validate and extrapolate the potential discriminatory ability of these findings for LC diagnosis.

## 3. Materials and Methods

### 3.1. Materials and Reagents

Sodium chloride (NaCl, 99.5%) was obtained from Panreac AppliChem ITW Reagents (Barcelona, Spain) to promote VOM’s salting-out. Hydrochloric acid (HCl, 37%), 3-octanol (99%, internal standard, 410 µg/L) and C7 to C30 alkane solution were obtained from Sigma-Aldrich (St. Louis, MO, USA). Ultrapure water from a Milli-Q system (Millipore, Bedford, PA, USA) was used to prepare the internal standard solution and HCl 5 M. The glass vials, SPME holder for manual sampling, and fibre were purchased from Supelco (Merck KGaA, Darmstadt, Germany). The SPME device included a fused silica fibre coating partially cross-linked with 50/30 μm divinylbenzene/carboxen/polydimethylsiloxane (DVB/CAR/PDMS), which was conditioned at 270 °C for 30 min before its use, according to the manufacturer’s instructions. Before each daily analysis, the fibre was conditioned for 10 min in the injector port to prevent carryover.

### 3.2. Sample Collection

Urine samples of LC patients and HCs were collected into 100 mL sterile, airtight polypropylene containers with an integrated transfer device (Greiner Bio-One GmbH, Kremsmünster, Austria) in the morning (without fasting), before tumour biopsy, at the Unit of Pneumology of SESARAM, EPERAM, Funchal. Samples were transported to the laboratory in a portable cooling box (±4 °C) suitable for biological specimens and processed within one hour of collection. Upon arrival, samples were centrifuged and aliquoted into 8 mL glass vials (to prevent repeated freeze-thaw cycles), and immediately stored at −80 °C until analysis, following the same procedure carried out with similar studies under development. Each aliquot was thawed only once before analysis. The study was performed in accordance with the principles contained in the Declaration of Helsinki and approved by the Ethical Committee for Health of SESARAM, EPERAM (S.24004894). All subjects signed an informed consent prior to participation. A match of age between LC patients and HCs was carried out. The study included 21 LC patients and 24 HCs, aged from 30–86 years (Table 2). The HCs were selected from blood donors of the Dr. Nélio Mendonça Hospital (HNM), eligible if they were 40–60 years of age or older and had no history of previously diagnosed cancer or other significant disease. Epigenetic factors and relevant information about the clinical characteristics of the study population were also considered in the current study. Among the LC cohort, three patients received systemic anticancer therapy before urine collection (two chemotherapy and one combined chemotherapy plus immunotherapy); one was subject to surgery followed by radiotherapy, and one to surgery. The remaining patients were treatment-naïve at the time of sampling. Detailed clinical information, including tumour stage, histological subtype, and treatment status, is provided in Table S1.

### 3.3. Urine VOM Extraction by HS-SPME

HS-SPME extraction was performed according to conditions previously optimised for similar studies [14]. For VOM extraction, 4 mL of urine, previously adjusted to pH 1–2 with 500 µL HCl (5 M), 0.8 g NaCl and 5 µL 3-octanol (16.4 µg/L), was placed in an 8 mL glass vial in a water bath set at 50.0 ± 0.1 °C under constant stirring (800 rpm). The SPME fibre was exposed to the headspace for 60 min. After extraction, the SPME fibre was collected and inserted into the injector port of the GC-MS instrument for 6 min at 250 °C, where the analytes were desorbed and transferred directly to the column. Each sample was analysed in triplicate.

### 3.4. GC-MS Analysis

VOM separation and identification were carried out using an Agilent Technologies 6890 N Network gas chromatograph system (Palo Alto, CA, USA) equipped with a SUPELCOWAX 10 fused silica column (60 m × 0.25 mm I.D. × 0.25 μm film thickness, SGE, Dortmund, Germany) connected to an Agilent 5975 quadrupole inert mass selective detector. The following oven temperature profile was set: 40 °C (2 min) followed by an increase in temperature until 220 °C, at a rate of 2.70 °C/min (hold for 5 min), giving a total run time of 73.67 min. The column flow was constant at 1.0 mL/min using helium (He, N60, Air Liquide, Porto, Portugal) as the carrier gas. The injection port was maintained at 250 °C and operated in the splitless mode. Regarding MS analyses, the operating temperatures of the transfer line, quadrupole and ionization source were 250, 150 and 230 °C, respectively. The electron impact mass spectra were recorded at 70 eV, and the ionization current was 10 μA. The data acquisition was performed in scan mode (30–300 *m*/*z*). The identification of VOMs was performed by comparing mass spectra with the data system library (NIST, 2005 software, Mass Spectral Search Program v. 2.2, Nist 2005, Gaithersburg, MD, USA), considering a minimum percentage match of 80%, Kovats index, and standard as available at the laboratory. To determine the Kovats index for the identified VOMs and allow their comparison with the Kovats index available in the literature for similar experimental conditions, the C7–C30 *n*-alkanes series was analysed under the same experimental conditions. All experiments were performed in triplicate, and the results were expressed as the mean relative peak area ± standard deviation.

### 3.5. Statistical Analysis

Statistical analyses were performed using MetaboAnalyst 5.0 [43]. The data matrix was normalised using a cubic-root transformation, followed by mean-centred scaling. Statistical comparisons between LC and HC samples were conducted using an unpaired Student’s *t*-test with Welch’s correction, which does not assume equal variances between groups. The normalised data were then processed using *t*-tests (*p*-values < 0.001). Based on the statistically significant VOMs, a multivariate analysis was performed using partial least squares discriminant analysis (PLS-DA). A heat map created using Euclidean correlation shows potential clustering patterns among the VOMs that were significantly altered in the different groups. Important PLS-DA model variables were selected using a variable importance in projection (VIP) score higher than 1, and the PLS-DA models were validated using 10-fold cross-validation (CV) and permutation tests (1000 random permutations of Y-observations).

## 4. Conclusions

The volatilomic urinary signature has the potential as a non-invasive tool in diagnostic investigations for distinguishing LC patients from HCs. Fifty-six VOMs, belonging to various chemical families, were observed with significant differences between groups. Increased levels of terpenoids and aldehydes were detected in LC patients, indicative of greater oxidative stress, inflammation and lipid peroxidation, whereas sulphur compounds and volatile phenols increased in HCs, indicating changes in sulphur amino acid metabolism and microbial activity linked to the pathophysiological state. Multivariate analysis using PLS-DA (R^2^ = 0.961, Q^2^ = 0.941, *p* < 0.001) and hierarchical cluster analysis provided a statistically robust separation between LC and HC groups, indicating distinct volatilomic signatures. Among the discriminative metabolites, octanal, dehydro-p-cymene, and 2,6-dimethyl-7-octen-2-ol emerged as potential biomarkers for early LC detection. Although the sample size is limited, the results reinforce the value of this preliminary study to validate the applicability of HS-SPME/GC-MS, in conjunction with up-to-date statistical tools, to study the volatilomics in human biological fluids. The present study should remain open to further research with other considerations, such as interindividual variability (e.g., age, gender, diet, smoking, microbiota, medication), clinical and environmental factors that would consolidate the robustness of the potential biomarkers. The ROC curve analysis was not performed due to the limited sample size and the exploratory design of the work. ROC evaluation will be included in future validation studies using larger and independent cohorts to assess the diagnostic performance of the identified VOMs. Furthermore, future studies should explicitly control for treatment status, enabling direct assessment of pharmacological effects on urinary volatilomic signatures, should be conducted on independent samples, larger populations, and should combine multi-OMICS approaches (proteomics, lipidomics, and metabolomics) for a comprehensive biological interpretation and to further consolidate the clinical and diagnostic significance of these findings.

## Figures and Tables

**Table 1:** Volatile organic metabolites identified in the HCs and LC patients using HS-SPME/GC-MS.

RT (Min)	VOMs	KI_cal_	KI_Lit_	Possible Origin	Frequency (%)		RPA Range	
					LC	HCs	LC	HCs
**Alcohols**								
36.50	3,7-Dimethyl-3-octanol	1434	1431	Exo (diet)	90	89	0.04–0.56	0.08–1.29
38.72	2,6-Dimethyl-7-octen-2-ol	1431	1439	Exo (diet)	90	90	0.05–1.14	0.09–2.09
39.74	2-Ethyl-1-hexanol	1453	1453	Endo/Exo (diet)	90	95	0.18–4.55	0.49–1.94
**Aldehydes**								
19.57	Hexanal	1063	1063	Exo (diet)	–	91	–	0.06–0.56
29.94	Octanal	1261	1263	Endo (syst)/Exo (diet, env)	90	–	0.07–0.33	–
35.33	Nonanal	1363	1365	Endo (bact, syst)/Exo (env, diet, bact)	98	–	0.11–1.92	–
41.60	Benzaldehyde	1491	1490	Endo (syst)/Exo (diet, env)	94	–	0.07–0.57	–
54.54	3,4-Dimethylbenzaldehyde	1783	1790	Exo (diet)	96	90	0.08–2.98	0.07–2.30
**Furanic compounds**								
10.13	Furan	809	802	Endo/Exo (diet, env)	86	91	0.08–0.67	0.09–1.04
11.74	2-Methylfuran	866	870	Exo (diet, env)	93	91	0.11–2.15	0.10–2.11
14.36	2,5-Dimethylfuran	943	943	Exo (diet, env)	93	95	0.11–1.10	0.14–0.93
17.44	2-Ethyl-5-methylfuran	1018	1018	Endo/Exo (diet)	86	93	0.05–1.08	0.09–0.57
27.39	2-Pentylfuran	1212	1213	Endo/Exo (diet)	90	93	0.01–0.33	0.02–0.28
28.12	DMMPTF	1227	1237	Unk	93	96	0.05–0.86	0.05–3.44
40.85	2-Acetylfuran	1476	1475	Exo (diet)	–	83	–	0.09–2.19
50.25	4,7-Dimethylbenzofuran	1677	–	Endo (syst)	94	93	0.05–3.45	0.06–1.20
**Ketones**								
18.24	3-Hexanone	1035	1037	Endo (syst)/Exo (diet)	91	90	0.03–0.45	0.03–0.39
21.81	4-Heptanone	1106	1118	Unk	91	91	0.14–77.98	0.12–25.60
24.10	2-Heptanone	1152	1151	Endo (bact, syst)/Exo (diet)	–	90	–	0.02–0.80
31.42	2,2,6-Trimethylcyclohexanone	1287	1284	Exo (diet)	91	89	0.07–2.93	0.06–0.47
52.97	Methylacetophenone	1746	1746	Exo (diet)	93	–	0.04–0.57	–
**Norisoprenoids**								
46.11	*β*-Ionone	1605	1622	Exo (diet)	90	–	0.05–0.51	–
52.17	Dehydro-ar-ionene	1726	1729	Exo (diet)	97	97	0.07–6.04	0.34–4.99
54.94	*β*-Damascenone	1793	1791	Exo (diet)	96	–	0.13–2.42	–
**Sulphur-containing compounds**								
8.72	Methanethiol	523	–	Endo (syst, bact)	83	90	0.19–5.12	0.23–1.51
19.20	Dimethyl disulfide	1056	1055	Endo (bact)/Exo (diet)	96	98	0.06–2.17	0.10–3.94
33.77	Allyl isothiocyanate	1333	1325	Exo (diet, env)	92	–	0.04–1.82	–
34.54	Dimethyl trisulfide	1348	1347	Endo (bact, syst)	–	94	–	0.05–1.47
**Terpenoids**								
24.35	1,4-Cineole	1157	1164	Exo (diet, env)	–	88	–	0.02–4.23
24.98	*α*-Terpinene	1168	1168	Exo (diet)	97	97	0.05–2.32	0.03–1.87
25.78	Limonene	1183	1183	Exo (diet)	96	93	0.04–1.15	0.03–0.30
26.41	Eucalyptol	1194	1194	Exo (diet)	–	98	–	0.02–0.40
28.44	δ-Terpinene	1233	1233	Exo (diet)	95	95	0.04–3.10	0.08–3.23
29.53	*p*-cymene	1253	1253	Exo (diet)	–	97	–	0.13–16.49
39.15	Cis-linalool oxide	1440	1464	Exo (diet)	90	90	0.04–0.37	0.08–1.20
41.59	Theaspirane	1491	1496	Exo (diet, env)	96	90	0.06–1.03	0.08–0.79
45.38	4-Terpineol	1571	1571	Exo(syst)	94	91	0.05–0.73	0.07–0.76
46.65	*β*-Cyclocitral	1596	1598	Exo(env)	90	–	0.05–0.98	–
46.67	Menthol	1597	1599	Exo(diet)	95	96	0.05–1.07	0.03–6.07
49.34	*α*-Terpineol	1660	1662	Exo (diet, env)	96	93	0.07–1.16	0.09–1.95
51.02	Phellandral	1698	1696	Exo (diet)	95	–	0.05–3.89	–
61.80	3,4-Dehydro-*β*-ionone	2049	–	Exo (diet, env)	97	95	0.13–2.10	0.20–1.61
62.82	Nerolidol	2076	2054	Exo (diet)	87	–	0.06–0.85	–
70.06	Cadalene	2165	2188	Exo (env)	99	98	0.07–3.02	0.11–1.98
**Volatile phenols**								
56.03	2-Methoxyphenol	1818	1816	Exo (diet, env)	–	94	–	0.04–1.68
61.30	Phenol	2036	2035	Endo/Exo (env)	91	97	0.10–2.00	0.18–2.58
64.12	*p*-Cresol	2104	2103	Endo (bact)/Exo	97	96	0.12–6.84	0.12–23.13
**Others**								
17.84	Toluene	1027	1027	Exo (env)	91	95	0.03–0.46	0.04–0.41
32.12	1,2,3-Trimethylbenzene	1299	1295	Endo/Exo (diet)	95	95	0.04–1.69	0.05–0.44
36.99	p-xylene	1394	1400	Exo (diet)	85	–	0.05–17.40	–
37.36	Dehydro-*p*-cymene	1401	1400	Exo (diet)	97	96	0.09–2.52	0.32–19.78
40.59	Durene	1470	1480	Exo (env)	97	–	0.05–1.10	–
48.52	Lavender lactone	1641	1635	Exo (diet)	–	94	–	0.06–0.68
51.64	Naphthalene	1713	1713	Endo/Exo (env)	95	–	0.06–0.62	–
62.21	2,6-Dimethylnapthalene	2060	2038	Exo (env, syst)	96	–	0.06–2.60	–
62.39	Octyl octanoate	2065	2020	Endo/Exo (diet)	86	–	0.05–2.04	–

Abbreviations: Bact—bacterial; Diet—dietary; DMMPTF—2,2-dimethyl-5-(1-methyl-1-propenyl)-tetrahydrofuran; Endo—endogenous; Env—environmental; Exo—exogenous; HCs—control group; KI_lit_—Kovat index literature for similar columns; KI_cal_—Kovat index calculated for a SUPELCOWAX 10 fused silica column (60 m × 0.25 mm I.D. × 0.25 μm film thickness); LC—lung cancer; RT—retention time; Syst—systemic; Unk—unknown;–—not detected.

**Table 2:** Demographic and clinical data of cancer-free controls (HCs) and lung cancer patients (LCs).

Group	HCs	LCs
Number of samples (F/M)	24 (6/18)	21 (2/19)
Age range (years)	30–86	41–82
Mean age ± SD (years)	60 ± 17	63 ± 8
Current smoker	7	14
Former smoker	2	3
Never smoker	15	2
Unknown	0	2

## Data Availability

The original contributions presented in the current study are included in the article and Supplementary Materials. Further inquiries can be directed to the corresponding author.

## Supplementary Materials

The following supporting information can be downloaded at https://www.mdpi.com/article/10.3390/ijms27020982/s1.

## Abbreviations

The following abbreviations are used in this manuscript:

CV	Cross validation
DVB/CAR/PDMS	Divinylbenzene/carboxen/polydimethylsiloxane
GC-MS	Gas chromatography mass spectrometry
HC	Health control
HS-SPME	Headspace solid phase microextraction
KI	Kovat index
LC	Lung cancer
PLS-DA	Partial least squares discriminant analysis
VOMs	Volatile organic metabolites
