# Supplementary material for: Urinary Volatilomic Signatures for Non-Invasive Detection of Lung Cancer: A HS-SPME/GC-MS Proof-of-Concept Study
Source: Int J Mol Sci. 2026 Jan 19;27(2):982. doi: 10.3390/ijms27020982 (PMC12842182; doi:10.3390/ijms27020982)
Supplement: Supplementary file 1 [file ijms-27-00982-s001.zip › ijms-4034230-supplementary.pdf]

Urinary Volatilomic Signatures for Non-Invasive Detection of Lung Cancer: A HS-SPME/GC-MS Proof-of-Concept Study

Patrícia Sousa [1], Pedro Berenguer [2], Catarina Luís [1], José S. Câmara [1,3], Rosa Perestrelo[1,*],

[1] CQM – Centro de Química da Madeira, Universidade da Madeira, Campus da Penteada, 9020-105 Funchal, Portugal

[2] Centro de Investigação Dra. Maria Isabel Mendonça, Hospital Dr. Nélio Mendonça, SESARAM, EPERAM, Avenida Luís de Camões, nº57, 9004-514 Funchal, Portugal

[3] Departamento de Química, Faculdade de Ciências Exatas e Engenharia, Universidade da Madeira, Campus da Penteada, 9020-105 Funchal, Portugal

SUPPLEMENTARY MATERIAL

Table S1

Tumour stage and morphology of each sample used in the LC group.

Sample	Tumour morphology[a]	Tumour stage	Treatment status[b]
**1**	Adenocarcinoma	IV	-
**2**	Adenocarcinoma	IV	Chemotherapy + immunotherapy
**3**	Squamous cell carcinoma	IIIA	-
**4**	Squamous cell carcinoma	IIIB	Chemotherapy
**5**	Adenocarcinoma	IV	-
**6**	Mixed morphology[c]	IV	Surgery + radiotherapy
**7**	Adenocarcinoma	IV	-
**8**	Carcinoma	IV	-
**9**	Squamous cell carcinoma	IB	-
**10**	Squamous cell carcinoma	IIIB	Chemotherapy
**11**	Adenocarcinoma	IV	-
**12**	Adenocarcinoma	IA2	Surgery
**13**	Mixed morphology[d]	IV	-
**14**	Mucoepidermoid carcinoma	IV	-
**15**	Squamous cell carcinoma	IV	-
**16**	Small cell carcinoma	IV	-
**17**	Small cell carcinoma	IIIB	-
**18**	Adenocarcinoma	IV	-
**19**	Squamous cell carcinoma	IIIC	-
**20**	Non-small cell carcinoma	IV	-
**21**	Adenocarcinoma	IV	-

[a] ICD-O-3 morphology codes were as follows: adenocarcinoma (M8140/3), basaloid squamous cell carcinoma (M8083/3), carcinoma (M8010/3), large cell neuroendocrine carcinoma (M8013/3), mucoepidermoid carcinoma (M8430/3), non-small cell carcinoma (M8046/3), small cell carcinoma (M8041/3), and squamous cell carcinoma (M8070/3). Non-specific morphologies (e.g., carcinoma and non-small cell carcinoma) were assigned by the pathologist when a more specific diagnosis could not be established.

[b] History of anticancer treatment before urine sample collection.

[c] Mixed morphology: adenocarcinoma (85%), large cell neuroendocrine carcinoma (10%) and basaloid squamous cell carcinoma (5%).

[d] Mixed morphology: large cell neuroendocrine carcinoma and squamous cell carcinoma (scarce).
